# Exploring CHEMeDATA. An interview with Damien Jeannerat: What is the CHEMeDATA movement?

**DOI:** 10.1002/ansa.202000041

**Published:** 2020-05-15

**Authors:** Damien Jeannerat, Paul Trevorrow

**Affiliations:** ^1^ NMRprocess.ch, Geneva, Switzerland and e‐Research/DLCM, University of Geneva Geneva Switzerland; ^2^ John Wiley and Sons Ltd., The Atrium Southern Gate Chichester UK

CHEMeDATA.org is an extension of the NMReDATA.org initiative, which emanated from the NMR community. In this community, there was a significant challenge in sharing assignment information. There are multiple databases where you can find diverse types of NMR spectra associated to compounds but no common system. Unlike, for example, X‐ray crystallography, where there is a habit of saving data in a format that can be used by everybody, in NMR there is no generally accepted practice or central database. There are rules regarding how to present data for publishing, but for sharing the spectra, for example, there is a lack of standardization. The NMReDATA initiative proposed a format for annotating NMR assignments. It makes the link between the information in the spectra (signals have chemical shifts, a structure, and, often, coupling constants) and the corresponding proton and carbons in the molecule. The challenge is to do this in a way that is not only human readable but also understandable by a computer. The NMReDATA initiative was a success. The community adoption was quite fast, and the most important part was the inclusion of the format by software developers. Now the main NMR processing software platforms enable export and storage in this new format. The next step is for chemists to provide these NMR records (or NMReDATA) as supplementary data when they submit articles for publication. Editors are encouraging it but the real pressure will come from the funding agencies requesting data sharing as part of researcher's data management plans.

When communicating about our NMR Initiative, I was visiting conferences and workshops on chemistry data. It turned out that the NMReDATA Initiative was often cited as a model. Considering the addition of other chemistry information using the same philosophy spontaneously defined CHEMeDATA. This broadening scope is now amplified through interaction with a number of people involved in standards with the International Union of Pure and Applied Chemistry (IUPAC). Collaboratively we have developed a proposal for the development of standard based on FAIR data management of spectroscopy data.^1^ So, CHEMeDATA is an extension rather than a generalization of what we have achieved for NMR.

An easy entrance into this jargon‐rich and easily overwhelmingly conceptual field is to focus on the archived files, often saved in the ZIP format. They are commonly used to transfer, as a single item, a set of files organized in a file folder tree. More and more, chemists submit supplementary information to publish in a chemistry journal and spontaneously use such an archive format. In the best cases, it includes spectroscopic data, spectra, spreadsheets, etc. in various formats. More‐often‐than‐not, chemists do not include the files corresponding to the molecular structures (.cdx, or .mol file) thinking wrongly that it is all too obvious what compounds they refer to. This anthropocentric idea neglects the fact that computers have a really hard time identifying molecules when complex figures from the article is the only source. The names of the compounds may be more straightforwardly accessible by a computer, but they maybe ambiguous, unprecise with respect to 3D information and lack a numbering of the atoms to make sense of the NMR assignment.

A second problem is that each researcher has his own idea on how to organize files in the folder tree of the zipped archive. The lack of systematic structure and the absence of metadata makes it impossible to automatically identify the relation between the files. The consequence is that in the favorable cases where archive files are found as supplementary data associated to journal article, on academic repositories, etc., we end up with zip files that are like a trunk you find in your attic – you may have a name on it but you have no list of what's inside. And when you open it is pretty messy – especially to a robot's “view.” In other words, metadata about the content and the relation between the elements are badly missing. The IUPAC project on the “Development of a Standard for FAIR Data Management of Spectroscopic Data," is addressing this problem and as an early implementation, the CHEMeDATA initiative will work on methods to identify and list the content of chemistry archive files and code the relations between their elements. In this manner, when we find a reference to chemistry data listed on an editor's site, or a university archive, one could determine its content without having to download the whole dataset and look into it. Just displaying a set of smart labels would make it clear to people and computer what is there. This system will also allow exchange between databases. For example, service specialized in, say, NMR, could collect the assignment of organic compounds, another, the IR spectra, provided the license permits it. This will make data become truly searchable – which is the basis of the first letter of the “FAIR” principles; “Findability.”^2^

## 1 WHAT ARE THE ADVANTAGES FOR RESEARCHERS?

The most obvious advantage for researchers is to increase the impact of their work. This also reflects on the visibility of the researcher. If the work that someone has performed can be used in different ways and be cited outside the circles of specialists it demonstrates the value of the research outside of the conclusion of the day. Let us consider the satellite images acquired for a weather forecast. They have an immediate use, for the weather forecast but a good archive of these same images can also be used to, say, model the maturity of crops as a function of sun exposure through the comparison of previous images. In fact, indirect uses turn out to often have longer lasting impact. What will mater in the future is very difficult to anticipate. Some may care only about the 13‐C satellites of your 1D proton NMR spectra or the unaccounted presence of compounds considered as “artifacts” by today's chemist. Maybe it is all under the noise level – is not an exciting prospect!? In short, the influence of the research can multiply and be used in secondary applications.

One can also mention of a more direct scientific benefit; consider that you specialize in a narrow field of a certain types of natural products. When analyzing your NMR spectra, you use computer‐assisted structure determination utilizing chemical shift predictions (among other tools). Under the hood, these software packages rely on complex algorithm “learning” from the NMR data available at the time of the software's release. If you work on a new class of compounds, prediction of chemical shifts may be substandard and cause concern due to the discrepancies with the experimental data. Indeed, predicted chemical shifts will have lower precision and accuracy when experimental data are lacking. If you publish your NMR data and the chemical shifts are well reported and computer readable, future releases of the software would include these new data in the training of their algorithm and YOU directly benefit the most of sharing your data because it improves the prediction of the type of compounds you study. In short, by sharing your data, you contribute to better chemical shift prediction tools – just to take one example. But the same will be true for NMR coupling constants and other spectroscopic information.^3^

## 2 WHAT DO YOU THINK ARE THE CHALLENGES FOR THE COMMUNITY ON ACHIEVING THESE GOALS?

The first one is a cautious approach to sharing data. Researchers are worried to have more work to do. However, when you think of the time it takes to save a file and drag and drop it in a submission system, provided the journal has the tools for doing this, it should be expedient. Some of the submission steps that are requested today should disappear making it easier for authors to deposit data. If you are asked to retype your name or re‐introduce the compound name you will get legitimately frustrated, drop the process and avoid using the system again. If journals could work to improve the user interface of input forms, connect them with automated checkers, compound name generators, etc, the deposit may become a benefit for the author. A lot of data could be checked in a fully automated manner, for example, the name, the mass, the formula of a compound could have its name confirmed by automatic comparison with the data generated from the structure – if a methyl is missing the author may be warned of an inconsistency in the data. In the future, these possible sources of error may also be avoided as the software generating the data could directly include the metadata about the molecule that's inside. Finally, the analytical platforms together with a good data‐management system would play an important role by providing researchers with pre‐validated data, allowing principal investigators to focus on writing the paper instead of double‐checking data. Again, generating chemistry data should be quick and convenient and become a benefit for the researcher.

Perhaps the less honorable reason for the reluctance to share data is that accessible data makes them prone to later scrutiny and retrospective discovery of errors. This also raises the question of the quality of the data – will all electronic data be correct? To this argument, the answer is simply that errors, or lack of details are there to be fixed and complemented. Think of how publications changed from paper‐only to today's ability to access articles within seconds from just about anywhere in the world. The future of chemistry data is unlikely to focus on errors. Workflow integrating and correcting any type of error will make the concern of an incorrectly assigned pair of carbons totally anecdotical – in fact such a tools already exist, they just need data, good or bad, to show their power.^4^, ^5^, ^6^, ^7^, ^8^, ^9^, ^10^, ^11^, ^12^, ^13^, ^14^, ^15^, ^16^, ^17^, ^18^ Destroying data, which is what occurs eventually if data are not shared at publication, is simply not the way scientific research expands. So, provide data, they will be re‐interpreted, if need be!

## 3 WHAT IS REQUIRED BY THE COMMUNITY TO ACHIEVE TO ACHIEVE THESE GOALS?

The community have to think on how to support the FAIR principles – in particular how to make the type of data we use every day more “Findable.” The keys points will be to define relevant “chemistry objects” in an electronic manner. The questions to answer are: What are their relevant parameters and their outcome. For example, one could say about an NMR spectrum, that the Larmor frequency is one of the parameters, a peak at a given chemical shift is an outcome. An NMR assignment should probably be a separate entity, where a peak at a given chemical shift is the input and the relation between the peak and a hydrogen atom in the chemical structure the outcome. NMR is well covered, but other spectroscopy and chemical information need some input. At present there are a relatively small group of people interested in Open Data. Sometimes the expertise is not in the right hands, for example, you have very good expert on metadata, who may have little exposure to the chemical problem. It is difficult to get the right combination of people. Currently we rely on the goodwill of a few who can make work‐case and demonstration examples. At some point, this work will need financial support to provide tools to evaluate recommended practice, generate and validate the newly defined chemistry metadata, etc. It will follow the needs of the community, update recommendation according to new possibilities, etc.

## 4 WHAT CAN WE LEARN FROM DISCIPLINES SUCH AS THE CRYSTALLOGRAPHIC COMMUNITY IN ACHIEVING THE MISSION OF THE CHEMEDATA MOVEMENT?

The crystallography community are inspiring because they succeeded very well. I do not think it is fair to say that it was easier for them but the kind of data they produce allowed a more direct access to the underlying information than, say, NMR. With the generation of three‐dimensional structures, there are fewer variables and less error‐prone human intervention. Access to the electronic information for the crystallographic community appeared early enough in the development of the web that having a centralized place made sense and this community have continued with that trajectory.^19^ Should all chemistry information be stored at a central database with the model of The Cambridge Crystallographic Data Centre (CCDC)? Right now, the tendency is clearly going towards multiple initiatives that coexists at multiple locations. My expectation is to see a range of databases and services with diverse shape and sizes. Some will have a broad range of data type – for example, Institution repositories or archived service such as Zenodo. Others will specialize in a specific field and include, say, the NMR spectra of organic compounds. Some will probably attempt to embrace the entire domain of chemistry. Such a service would probably only include metadata and forward the user to the actual location of the data. They may be used as search tools and links towards horizontal and vertical sources. Others may focus on the chronological evolution of chemistry information. Indeed, correcting and complementing data require a powerful versioning system allowing to archive, rank and evaluate contributions by possibly very diverse contributors – including robots.

## 5 WHAT DO YOU SEE AS THE NEXT STEPS FOR THE CHEMEDATA MOVEMENT?

I think the next step is to communicate the need to simply provide chemistry data at the time of publication. One should not wait for perfectly annotated data to start the good habit of sharing chemistry data. Sure, the metadata associated with the chemistry data will greatly increase visibility and reusability but future progress in artificial intelligence may be quite able to fill the gap. In parallel we should be cognizant of endeavors in this space by different initiatives undertaken by other fields of science. This will help determine what is viable for the chemistry community and to see what recommendations IUPAC can synthesize from this priori. The biggest error would be to do something completely new; we should take the existing, push towards the broadly accepted format and see how the community reacts. We should keep in mind that companies will only implement recommendations involving simple changes to their products unless the new data opens now business opportunities.

## 6 HOW CAN WE AS A COMMUNITY, GET INVOLVED?

First, better organize your data to prepare them for sharing them when needed. Don't forget the structure files – don't worry about redundancy and uneven quality of the data – computers don't determine the fate of your soul. If your favourite journals are not requesting data, store them on third party archive services – it may well be that you will be the first to use them a few years later after you have changed computer four times and locations three! One last thing: If you ever post one of these unusable pdfs including images of your NMR spectra (because this is what people do) please include the crude spectra from the NMR instrument. The later are the file structures with numbers including fid, parameters, etc. Secondly, include the results of your hard assignment work! So, provide the file generated by your favorite NMR assignment software – don't waste it!
